# Clinical heterogeneity of low flow spinal arteriovenous fistulas; a case series

**DOI:** 10.1186/s12883-021-02394-3

**Published:** 2021-09-21

**Authors:** D. Krishnan, S. Viswanathan, N. Rose, H. S. N. Benjamin, A. M. Ong, F. L. Hiew

**Affiliations:** 1 Department of Neurology, Hospital Kuala Lumpur, Jalan Pahang, 50586, Kuala Lumpur, Malaysia; 2 Department of Radiology, Hospital Kuala Lumpur, Kuala Lumpur, Malaysia

**Keywords:** Spinal arteriovenous fistula, Low-flow, Clinico-radiological mismatch, Case series

## Abstract

**Background:**

Spinal AVF (SAVF), a potentially treatable cause of myelopathy, remains a challenging diagnosis. Its rarity and non-specific imaging findings often result in misdiagnosis despite a high index of clinical suspicion. The classically described high T2 signal in the spinal cord or prominent vascular flow voids in the intradural space were not infrequently missed on initial imaging, only to be picked up at follow-up imaging after progression of symptoms. Additionally, small sized fistulas(< 1 mm) and SAVF involving less frequent locations like the craniocervical junction in a patient presenting with paraplegia further complicates the diagnosis. On rare occasions, acute atypical presentation following a surgery adds to the conundrum. Definite diagnosis with spinal angiography, the gold-standard modality requires the expertise of highly skilled interventionists which may otherwise lead to false negative findings. We describe four SAVF patients with unconventional presentations, highlighting less described clinical findings.

**Case presentation:**

First was a 50-year-old man presented with spastic paraparesis and was found to have an AVF at the cervical region arising from the vertebral artery. Second, a 45-year-old man with acute paraplegia post-operatively, initially treated for a transverse myelitis before lumbar region AVF was detected. Thirdly, a 27-year-old man presented with subacute lower thoracic myelopathy and deteriorated after corticosteroid treatment. The last patient, who initially appeared to have conus medullaris/cauda equina syndrome had a SAVF at the mid thoracic level. Presentation varied with some exhibiting acute deterioration mimicking other spinal cord pathology such as inflammatory disorders. All patients eventually underwent endovascular treatment with successful embolization of SDAVF. None of them exhibited further neurological deterioration after embolization.

**Conclusion:**

Successful treatment of SAVF is possible provided the diagnosis is made early, allowing timely intervention. Certain clues may aid the diagnosis. Firstly, arteriovenous fistula can be located distant to the clinical localization of myelopathy resulting in the unexpected longitudinally extensive spinal cord signal change. This clinical-radiological discrepancy can be a useful clue in diagnosing SAVF. Secondly, an acute myelopathic presentation immediately post-surgery may be related to SAVF. Other SAVF feature of note includes progressive myelopathy mimicking immune-mediated myelitis among young adults below 30 years of age refractory to immune therapy.

## Background

Spinal arteriovenous fistulas (SAVFs) is a rare but treatable cause of chronic myelopathy as a result of progressive spinal venous hypertension. Available evidence suggests that SAVFs are acquired lesions due to obstructed spinal venous drainage although the actual etiology remains unclear. Despite increasing recognition of this disease and advancing surgical skills over the recent years, the diagnosis of complex SAVFs involving the low-flow fistulas especially at less common sites with atypical clinical presentation and non-specific spinal cord imaging appearance remains a challenge. SAVFs involving the craniocervical junction are rare, but are the most dangerous among all due to their potential significant brainstem oedema secondary to venous congestion. This can lead to an increased risk of subarachnoid or posterior fossa haemorrhage [1, 2]. Very often, this variant of SAVFs is misdiagnosed even among experienced clinicians [3]. Unlike the high-flow pattern of other spinal vascular malformations, in which enlargement of perimedullary and/or epidural veins are readily identified by MRI imaging, the low-flow SAVFs are often missed or misdiagnosed due to a heterogenous spectrum of clinical presentation and non-specific appearance on imaging [4]. Common locations for low-flow SAVFs are perimedullary, epidural and dural [5]. A recent study on the sources of radiological error in diagnosing dural SAVFs found that over half of the radiologically confirmed cases were misinterpreted on initial MRI despite presence of typical imaging characteristics such as dilated veins, increased T2 signal and enlarged spinal cord segments [6]. There is no denying that the diagnostic process of this rare entity requires more than the standard routine imaging sequences.

We report 4 cases of low-flow SAVFs with longitudinally extensive, multi-segment lesions involving more than one spinal cord region. The atypical clinical course and imaging characteristics discussed unveils the paradigm of clinical heterogeneity in a single entity.

The clinical characteristics, chronology of events and outcome of patients are summarised in Table 1.

**Table 1:** Clinical characteristics, chronology of events and outcome of patients

Patient	1	2	3	4
Age at presentation (years)	50	45	27	56
Sex	Male	Male	Male	Male
Symptoms onset to presentation	3 months	1 day	2 weeks	5 months
Presentation	Numbness and weakness of both LL	Numbness and weakness of both LL	Numbness and weakness of both LL	Acute urinary retention, back pain, numbness and weakness of LL
Antecedent event	None	Haemorrhoid surgery	None	None
Clinical deficits on presentation				
Motor deficit	LL spastic paraparesis MRC 3	LL spastic paraparesis MRC 1	LL spastic paraplegia MRC 4	LL spastic paraparesis MRC 3
Sensory level	T6	L1	T12	L4
Bladder involvement	Yes	Yes	Yes	Yes
Bowel involvement	Yes	No	Yes	No
Clinical deficits at maximum severity				
Motor score (MRC)	MRC 0/5	MRC 0/5	MRC 0/5	MRC 0/5
Functional score (MRS)	MRS 5	MRS 4	MRS 3	MRS 4
MRI scan findings				
Spinal cord regions	Craniocervical	Mid thoracic to upper lumbar	Mid thoracic to upper lumbar	Mid thoracic to upper lumbar
Abnormal signals	Intramedullary hyperintensities extending from the medulla to the C7 cord	Intramedullary T2/FLAIR hyperintensity extending from T6 to L2	Intramedullary T2/Flair hyperintensity from T7 to L1	Intramedullary T2 hyperintensity from T6 to L1 with contrast enhancement
MRI scan findings on maximum disease severity	Centrally hyperintense lesion extending from the pons down to T1 with cord expansion. Anterior serpinginous flow void signals at the upper cervical region	Intramedullary T2/Flair hyperintense lesion from T6 up to L2 with cord expansion and minimal contrast enhancement	Intramedullary T2/FLAIR hyperintense lesion with patchy enhancement extending from T7 to L1 spinal cord	Intramedullary T2 hyperintensity from T4 cord to the conus medullaris with dilated spinal veins seen most prominently from T10 to L1
Spinal angiogram	Dural fistula at left C1 level with a likely feeding artery from meningeal branch of left vertebral artery	Suspicious spinal dural arteriovenous fistula (SDAVF) at the L2 region	Dural fistula at left L2 lumbar artery	Initial negative but repeated showed SAVF from left 12th posterior intercostal arteries and L1 lumbar artery
Response to steroid	Paradoxical worsening	Improvement	Partial response initially with paradoxical worsening	Paradoxical worsening
Outcome after embolisation	No improvement	No improvement	Improvement	No improvement
Motor score (MRC)	LL MRC 0 UL MRC 4	LL MRC 0 UL MRC 5	LL MRC 4	LL MRC 1
Functional score (MRS)	MRS 4	MRS 4	MRS1	MRS 4

*Abbreviations*: *MRC* Medical research council, *MRS* Modified Rankin Scale, *LL* lower limbs, *UL* upper limbs, *SAVF* spinal arteriovenous fistula, *SDAVF* spinal dural arteriovenous fistula

## Case presentation

### Case 1

**Fig. 1:**
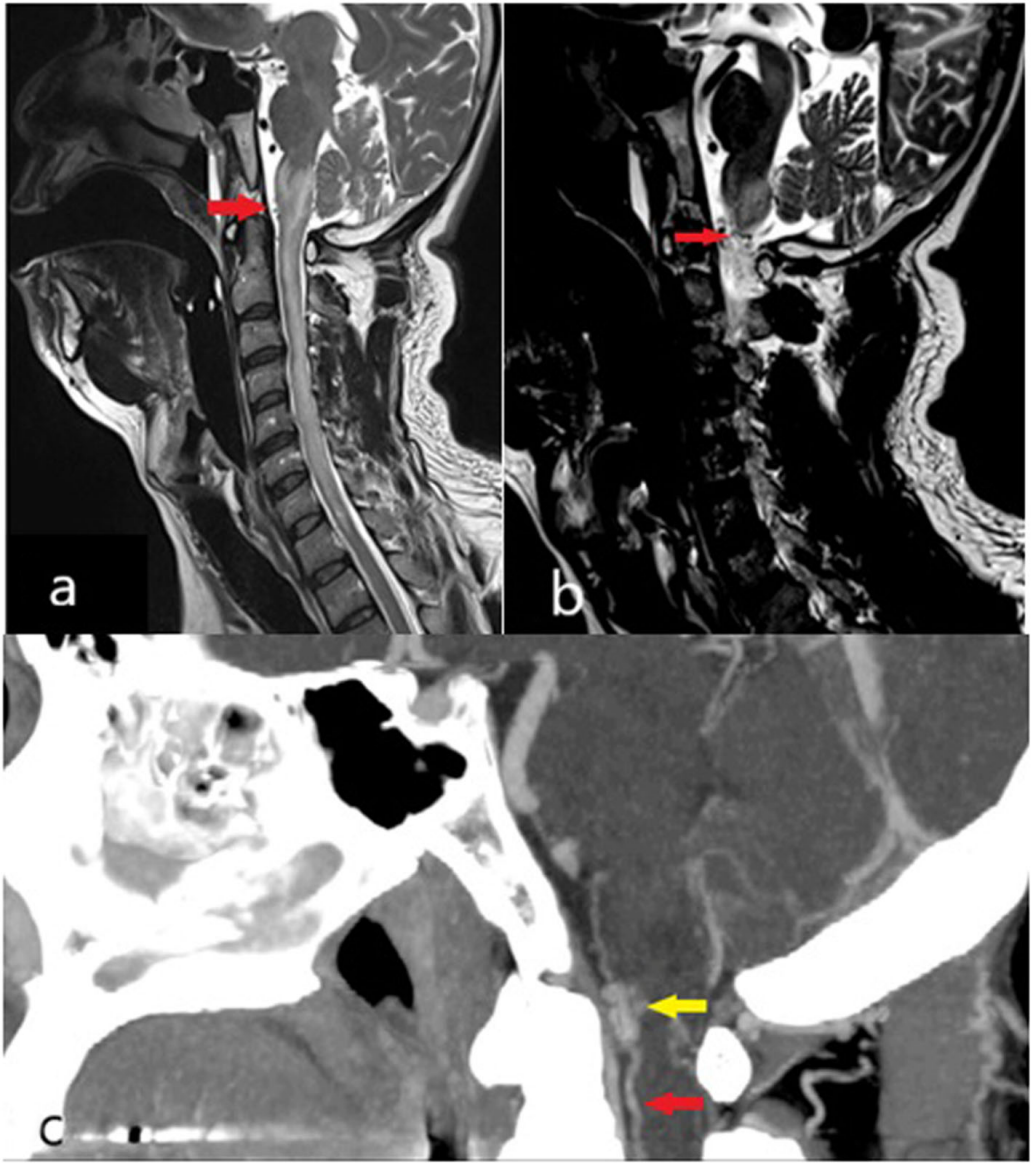
**a** and **b** MRI of patient 1 demonstrated longitudinal extensive T2 hyperintensity from medulla to C7 spinal cord level with dilated perimedullary veins anterior to the cord at the cervical region (red arrow). **c**. CT angiogram demonstrated arterio-venous fistula (yellow arrow) at C1 level and the anterior dilated perimedullary veins (red arrow)

A 50-year-old man presented with progressive spastic lower limb paraparesis over 3 months associated with urination retention. Neurological examination revealed a sensory deficit below the T6 dermatome. MRI demonstrated intramedullary T2 hyperintensities extending from the medulla to C7 spinal cord with heterogenous contrast enhancement and cord expansion. Despite being negative for serum Aquaporin 4 (AQP4) IgG and cerebrospinal fluid (CSF) oligoclonal band (OCB), he was treated as immune-mediated longitudinal extensive transverse myelitis (LETM) with intravenous (IV) Methylprednisolone. Two days later, his weakness progressed to quadriparesis. He subsequently received 5 sessions of therapeutic plasma exchange (TPE), but showed no response. A repeat MRI/MRA of the spine revealed extension of the hyperintense lesion from pons to T1 level with cord expansion and anterior serpinginous flow void signals *(*Fig. 1a and b). Spinal angiogram confirmed a SAVF at left C1 level with a feeding artery from the meningeal branch of the left vertebral artery. Endovascular embolisation was attempted but was incomplete due to technical difficulties. Imaging was repeated in view of poor neurological recovery. MRI done a year later demonstrated worsening of SAVF with new abnormal dilated epidural vessels at C6 to T6 level. Despite successful surgical ligation, there was no satisfactory clinical improvement and his functional outcome remained poor.

### Case 2

**Fig. 2:**
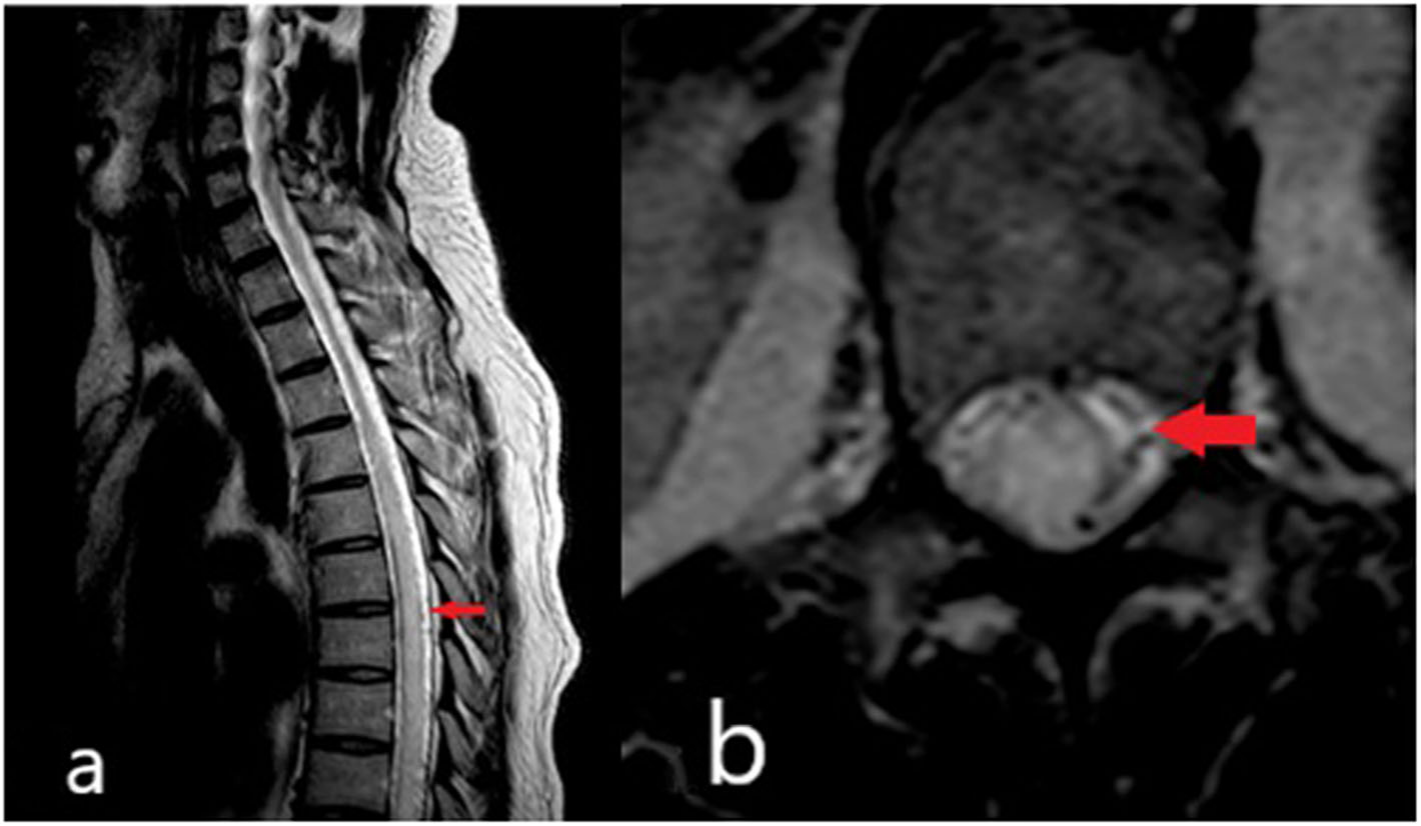
**a** and **b** MRI of patient 2, showing the suspicious dilated vessels at the posterior aspect of the thoracic cord (red arrow)

A 45-year-old gentleman with no prior illness was admitted for hemorrhoidectomy under general anesthesia. Immediately following surgery, he complained of bilateral lower limb weakness and numbness with difficulty passing urine. Clinical assessment revealed paraparesis and loss of sensation with sensory level below the L1 dermatome. MRI of the thoracic and lumbosacral spine revealed an intramedullary T2 hyperintense lesion extending from T6 to L2 vertebra level with cord expansion and minimal contrast enhancement. He was treated as LETM with 3 days of IV Methylprednisolone and was subsequently discharged. Despite initial improvement in lower limb power after 2 weeks, he remained dependent on a urinary catheter and required laxatives to aid bowel opening. His lower limb weakness worsened 1 month later. An urgent MRI of the whole spine showed similar findings. In addition to second course of IV Methylprednisolone, he received 5 cycles of TPE but showed minimal improvement. A year later, he presented again with worsening paraparesis. Repeat MRI revealed a lesion suspicious of SAVF at the L2 region *(*Fig. 2a and b). This was confirmed with a spinal angiography after which he underwent successful embolisation. Despite no further relapses, his neurological recovery was poor.

### Case 3

**Fig. 3:**
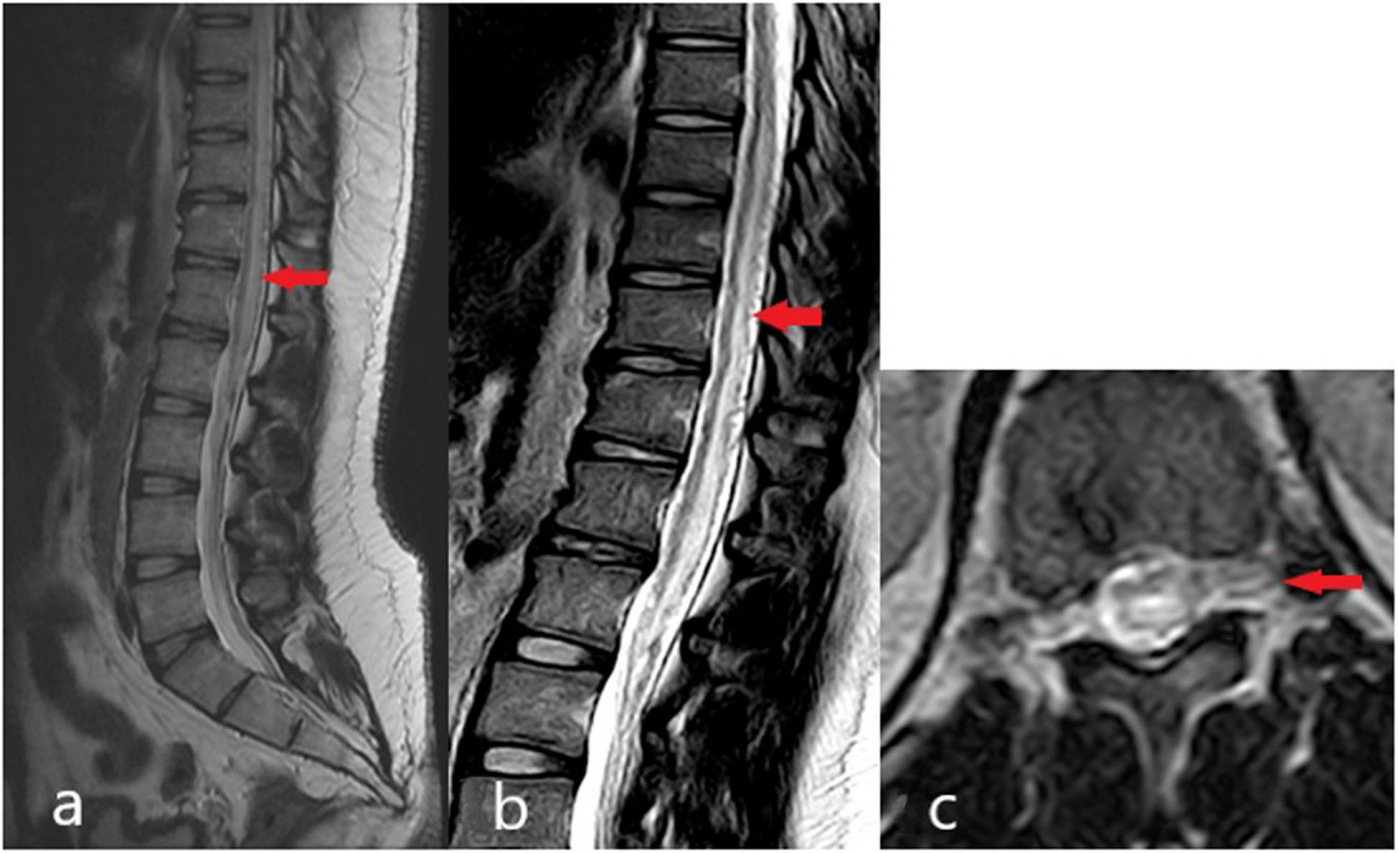
**a** and **b** MRI of patient 3 demonstrated T2 hyperintense intramedullary lesion with patchy enhancement extending from T7 to L1 (red arrows) and features suspicious of SDAVF with the dilated vessels seen at the thoracolumbar spinal cord (red arrows). **c**. MRI which clearly shows the dilated vessels and the feeding vessel (red arrow)

A 27-year-old gentleman presented with acute bilateral spastic paraparesis and bowel/bladder dysfunction with a sensory level at T12. He had a similar presentation 2 years ago and was treated as immune-mediated LETM in another institution. Following a course of IV methylprednisolone and intravenous immunoglobulin (IVIG), he had partial recovery and was able to ambulate despite a right foot drop and required intermittent bladder catheterization. Upon admission to our center, an urgent MRI of the spine reported a T2 hyperintense intramedullary lesion with patchy enhancement extending from T7 to L1. He rapidly deteriorated and became paraplegic over the next few days despite retreatment with IV Methylprednisolone. MRI images were revisited, with identification of flow void signals at the posterior aspect of the cord from T6 to L2 *(*Fig. 3a, b and c), which later confirmed a SDAVF at the L2 region on spinal angiogram. The patient underwent successful endovascular embolisation and showed good neurological recovery.

### Case 4

**Fig. 4:**
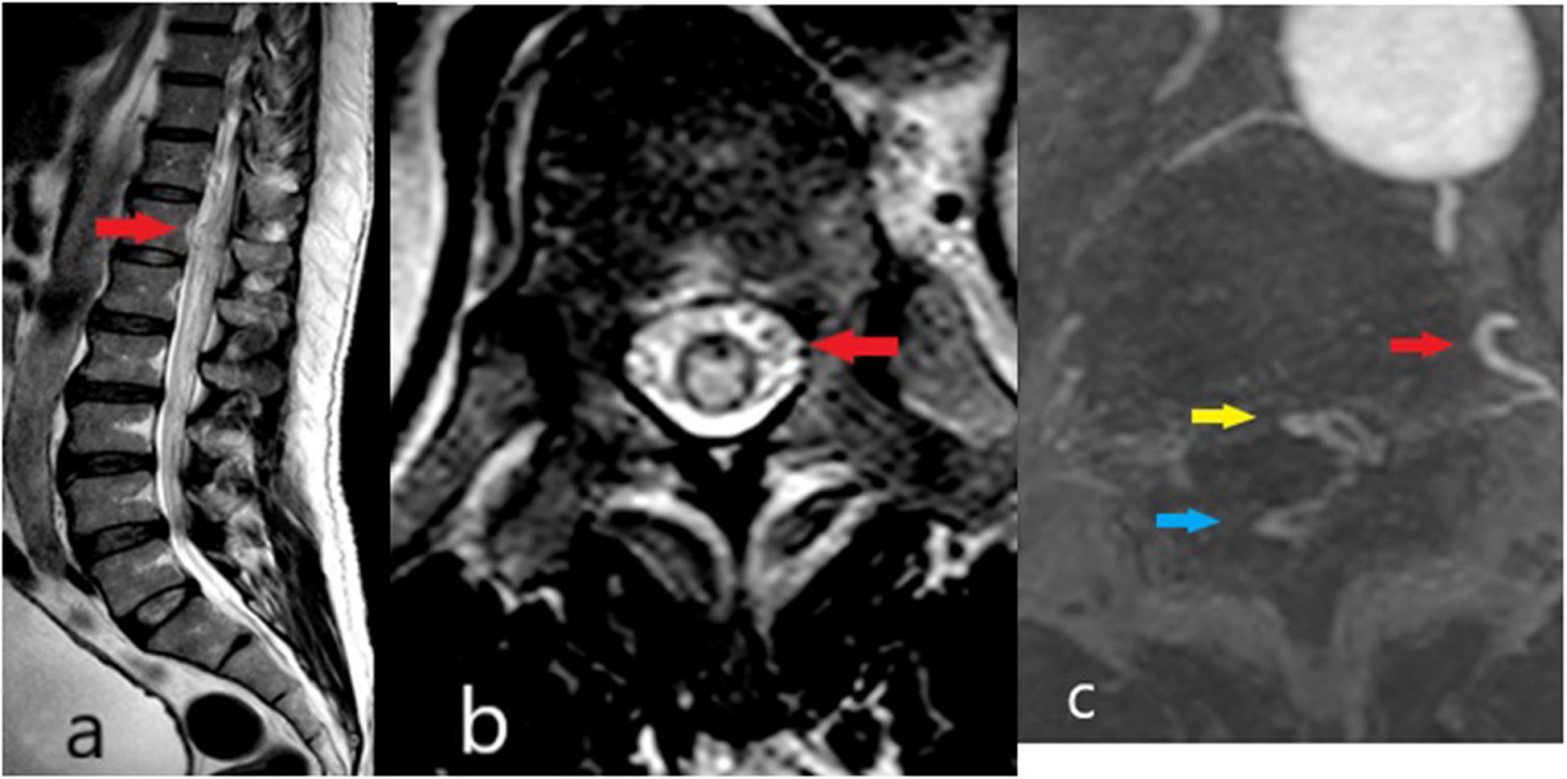
**a** and **b** MRI spine of patient 4 showing the suspicious dilated vessels at the thoracolumbar cord (red arrow). **c**. CT angiogram (row below) demonstrated the feeding vessel (red arrow), dural AVF (yellow arrow) and the dilated perimedullary vein (blue arrow)

A 56-year-old gentleman presented with a month history of lower back pain followed by paraparesis. Prior to that, he had an episode of acute urinary retention requiring temporary indwelling catheterization. MRI of the whole spine done at another center concluded degenerative spine disease. Upon assessment, sensation was reduced below the L4 dermatome. Repeat MRI showed a longitudinal extensive T2 hyperintense contrast enhancing intramedullary lesion from T6 to L1. His lower limb weakness deteriorated soon after the first dose of Methylprednisolone. Despite strong clinical suspicious of SAVF, his spinal angiogram was negative for this. He received a course of IV Methylprednisolone followed by plasmapheresis but showed no remarkable improvement. Within a year, he was readmitted twice for similar relapses and responded poorly to IV methylprednisolone. Repeated MRI spine demonstrated a worsening contrast enhanced T2 hyperintense lesion involving T4 to the conus medullaris with dilated spinal veins seen most prominently from T10 to L1 level *(*Fig. 4a and b). A second spinal angiogram confirmed a SAVF with feeders from the left 12th posterior intercostal arteries and L1 lumbar artery. Despite successful embolisation, his neurological recovery was poor.

## Discussion and conclusion

We described 4 cases of low-flow dural SAVFs with unique heterogeneous clinical spectrums. We discuss 4 important but less-described aspects of clinical practice in the management of SAVFs.

Firstly, the clinical presentation of the low-flow variant of SAVFs correlated poorly with radiological findings. Myelopathy and sensory deficit inaccurately predicted the extent of spinal cord intramedullary T2 signal and the level of SAVF. This was thought to be due to the unequal distribution of venous outflow channels along the spinal cord, with the lower thoracic region having relatively fewer venous outflow channels compared to the cervical region. This results in venous congestion being transmitted in a caudocranial direction, explaining the more severe myelopathy symptoms correlating with a more distal region of the MRI signal [7, 8]. This also explains why majority of the SAVFs involve the thoraco-lumbar region. Similarly, the rate of clinical progression and severity are independent of the length of abnormal spinal cord signal. Our patient with high cervical SAVF from a feeding artery that originated from the meningeal branch of the left vertebral artery was completely lacking brainstem signs as well as the commonly reported subarachnoid hemorrhage or intraparenchymal bleed within the brainstem [9]. The actual reason behind this is unclear. One study described five different types of craniocervical junction AVFs (Dural, Radicular, Epidural with pial feeders, Epidural and Perimedullary) based on location of the shunt point. The authors inferred that radicular type is more commonly associated with SAH besides other factors such as aneurysm of the feeder artery and anterior spinal artery as the feeder. All these features were not present in our patient [10]. The extent of spinal cord ischemia resulting in infarction likely plays a more important role than spinal cord oedema which is largely reversible [11]. From a recent analysis, symptoms of brainstem dysfunction are very rarely reported, occurring in only 3.3% of patients with craniocervical junction SAVFs [12]. This highlights the importance of careful imaging as a SAVF in a particular location may produce symptoms and signs further away as a result of intramedullary venous oedema [13]. This clinical-radiological discrepancy/mismatch may be an important clue to differentiate SAVFs from other causes of myelopathy.

Secondly, increased venous hypertension in low-flow SAVF reflects the underlying dynamic vascular pathophysiology of this disorder. Although the underlying aetiology and pathophysiology of SAVF is not perfectly understood, we know that arterialization of the venous system diminishes the arteriovenous pressure gradient and leads to a decreased drainage of normal spinal veins, resulting in venous congestion [7]. Therefore, any disruption around this arteriovenous system may result in SAVFs. In relation to this, we observed 2 unique events among our patients that could have led to the development of myelopathy. One of our patients developed symptoms following hemorrhoidectomy. To our knowledge, acute presentation post-operatively, and in our case, following hemorrhoid surgery, has not been reported. The pathogenesis of hemorrhoids is thought to be due to abnormal distension of the arteriovenous anastomoses within the hemorrhoidal cushions, in which part of the surgical treatment involves the ligation of hemorrhoidal arteries [14, 15]. It is still unclear if hemorrhoidectomy could affect the hemodynamics within the inferior vena cava (IVC) and the venous system, resulting in increased venous pressure at the spinal venous plexus contributing to an acute presentation in a patient with a pre-existing subclinical SAVF. Dural SAVF reported following spinal surgery such as correction of scoliosis and microdiscectomy for radiculopathy, further supports the postulation of an acquired traumatic or inflammatory mechanism [16, 17]. Another patient of ours developed extensive dilated epidural vessels from C6 to T2 following an incomplete embolization of the left vertebral artery SAVF. Although this occurred after a year, it was not unusual. In majority of the patients, SAVFs, symptoms are usually slowly progressive over many months to years. Elswick and colleagues reported a case of dural SAVF that developed 5 years after thoracolumbar fusion for scoliosis [16].

Thirdly, majority of patients with SAVFs have MRIs that show either longitudinally extensive intramedullary T2 hyperintense signal, flow voids or cord expansion. The probability of one of these MRI changes occurring in a patient is reported to be as high as 100% [18]. Despite that, these features are frequently missed due to its rare occurrence [19]. Spinal angiogram is considered the gold standard investigation to confirm SAVFs [20]. However, no imaging modality has perfect sensitivity and specificity. A false negative spinal angiogram may further complicate clinical judgement. This could be due to misinterpretation of images, technical difficulties especially when involving small SAVF < 1 mm or limited spinal segments on angiogram study [3, 13, 20]. Highly specific angiographic techniques performed by experienced interventional specialists and meticulous analysis of angiographic images are essential for accurate diagnosis and identification of the location of the fistula [3, 4, 20].

Lastly, low-flow SAVF is typically diagnosed among older patients presenting with a progressive myelopathy secondary to spinal venous hypertension. The diagnosis is extremely uncommon in the younger population and is rarely discovered before the age of 30, although one of our four patients was 27 years old. It is estimated that disease onset before 30 of age constitute less than 1% of the patients [7]. We could not identify any specific history or precipitating factors in this patient, but in comparison to the other 2 patients with relatively similar thoracolumbar SAVF presentation at maximum severity, the outcome post-embolization was far better. Although his presentation was relatively early within 2 weeks of symptoms onset, which has been associated with better long-term outcome [13, 21], similar outcome was not observed in case 2 with symptom onset post-hemorrhoidectomy. From a recent analysis, patient’s age and neurological status at diagnosis had no influence on the long-term outcome. This was in contrast to the conventional assumption that younger patients have better recovery potential and likewise, the more severe the neurological deficit, the poorer the recovery [21]. These observations reflected the ongoing lack of understanding of the disease.

Neurosurgery and endovascular embolization have been employed in the management of SAVF. Endovascular modality has been the traditional first line approach in our institution, possibly due to the limited expertise and risks involved with open spinal surgery. However, several studies and meta-analyses over the years have consistently shown better success and lower recurrence rates with neurosurgery compared to endovascular treatment; although the outcome of the latter has improved over time [22].

We described the atypical presentation and clinical heterogeneity seen among patients with low-flow SAVFs. Careful recognition of these uncommon features such as prominent high craniocervical lesions without brainstem signs, symptom onset in association with a recent surgery, false negative spinal angiogram, and SAVF in young adults may change the clinical course of the disease and improve functional prognosis.

## Data Availability

All relevant data are available at the Neurology clinic, Kuala Lumpur General Hospital.

## Abbreviations

AQP4: Serum Aquaporin 4
CSF: Cerebrospinal fluid
IVIG: Intravenous immunoglobulin
IVC: Inferior vena cava
LETM: Longitudinal extensive transverse myelitis
MRI: Magnetic resonance imaging
OCB: Oligoclonal band
SAVFs: Spinal arteriovenous fistulas
TPE: Therapeutic plasma exchange
